# Decolonizing drug-resistant *E. coli* with phage and probiotics: breaking the frequency-dependent dominance of residents

**DOI:** 10.1099/mic.0.001352

**Published:** 2023-07-07

**Authors:** Jessica H. Forsyth, Natalie L. Barron, Lucy Scott, Bridget N. J. Watson, Matthew A. W. Chisnall, Sean Meaden, Stineke van Houte, Ben Raymond

**Affiliations:** ^1^​ Centre for Ecology and Conservation, University of Exeter, Penryn, TR10 9FE, UK; ^†^​ Present address: Division of Evolution, Infection and Genomics, School of Biological Sciences, Faculty of Biology, Medicine and Health, The University of Manchester, Manchester M13 9PT, UK; ^‡^​ Present address: Department of Biology, University of York, Wentworth Way, York, YO10 5DD, UK

**Keywords:** AMR, antagonistic competition, drug resistance, frequency dependence, phage therapy, resistance management

## Abstract

Widespread antibiotic resistance in commensal bacteria creates a persistent challenge for human health. Resident drug-resistant microbes can prevent clinical interventions, colonize wounds post-surgery, pass resistance traits to pathogens or move to more harmful niches following routine interventions such as catheterization. Accelerating the removal of resistant bacteria or actively decolonizing particular lineages from hosts could therefore have a number of long-term benefits. However, removing resident bacteria via competition with probiotics, for example, poses a number of ecological challenges. Resident microbes are likely to have physiological and numerical advantages and competition based on bacteriocins or other secreted antagonists is expected to give advantages to the dominant partner, via positive frequency dependence. Since a narrow range of *Escherichia coli* genotypes (primarily those belonging to the clonal group ST131) cause a significant proportion of multidrug-resistant infections, this group presents a promising target for decolonization with bacteriophage, as narrow-host-range viral predation could lead to selective removal of particular genotypes. In this study we tested how a combination of an ST131-specific phage and competition from the well-known probiotic *E. coli* Nissle strain could displace *E. coli* ST131 under aerobic and anaerobic growth conditions *in vitro*. We showed that the addition of phage was able to break the frequency-dependent advantage of a numerically dominant ST131 isolate. Moreover, the addition of competing *E. coli* Nissle could improve the ability of phage to suppress ST131 by two orders of magnitude. Low-cost phage resistance evolved readily in these experiments and was not inhibited by the presence of a probiotic competitor. Nevertheless, combinations of phage and probiotic produced stable long-term suppression of ST131 over multiple transfers and under both aerobic and anaerobic growth conditions. Combinations of phage and probiotic therefore have real potential for accelerating the removal of drug-resistant commensal targets.

## Data Summary

Two phage genomes have been submitted to the European Nucleotide Archive as *Escherichia* phage vB_EcoP-CC1 and vB_EcoP-CC2 with accession numbers GCA_949752765.1 and GCA_951812185.1 and study accession number PRJEB59323. Experimental data are available using the following permanent doi at Open Research Exeter https://doi.org/10.24378/exe.4684.

**Impact Statement**

New tools for dealing with the ongoing antimicrobial resistance crisis are desperately needed. Antibiotic prescribing can leave a legacy of resistant bacteria that can persist in the gut. These infections are not always harmful but are known to increase the risk of difficult-to-treat infections for up to a year after antibiotics have been given. Dislodging these resistant bacteria can therefore have a number of health benefits for individuals and could reduce the burden of resistance in targeted populations. Resident bacteria can be difficult to dislodge because they will have a numerical advantage when compared to any newly ingested bacteria such as probiotics. Bacteria-specific viruses or phage have also been investigated as potential tools for dislodging resistant bacteria but can fail to work due to the evolution of resistance to phage infection. This study aimed to find a new means of suppressing a very common but harmful lineage of antibiotic-resistant *E. coli *. In particular, we tested, in laboratory conditions, whether combinations of a host-specific phage and competing probiotic *E. coli* were more effective than phage or probiotic alone. Under a range of growth conditions, we showed that combinations of phage and competing probiotics were much more effective than either treatment alone. If we can demonstrate the safety and efficacy of these combinations in live human infections in future studies this could establish a new tool for suppressing antibiotic-resistant bacteria before or after medical treatment.

## Introduction

Antibiotic resistance in commensal bacteria that can cause hospital-acquired infections is a significant challenge for healthcare [1]. These organisms are exposed to selective pressure from antibiotics regardless of whether they are the target of therapy. Niches such as the gut provide ample opportunities for horizontal gene transfer and the acquisition of low-cost resistance, most often encoded by plasmids [2]. Commensal bacteria are commonly Gram-negative, which comprise some of the most challenging multidrug-resistant (MDR) bacteria in terms of treatment [3, 4]. Antibiotic prescribing incurs long-term risks of harmful drug-resistant infection and this is at least partially thought to be the result of selection on commensals [5, 6]. Asymptomatic carriage of MDR bacteria has several clinical consequences, including increased risks during routine surgical interventions and increased risk of drug-resistant infections [7]. For *Escherichia coli* in particular, colonization of the intestine is believed to be a prerequisite for the development of urinary tract infections (UTIs) and bacteraemia [8–10].

One approach to widespread colonization by antibiotic-resistant bacteria is selective removal before they can cause disease. While this is challenging for many species, ‘decolonization’ is already in clinical practice in intensive care and may become increasingly important for patients requiring interventions such as bowel surgery [11]. Of all the Gram-negative commensals, *E. coli* probably presents the best target for a decolonization approach that could be scaled up to a population-level intervention. This is because a large majority of the pathogenic and MDR infections caused by *E. coli* derive from a narrow range of genotypes. Extra-intestinal pathogenic *E. coli* (ExPEC) are the leading cause of human extra-intestinal infections globally, yet only a small subset of ExPEC lineages cause the vast majority of infections [12]. One such lineage is sequence type (ST) 131 [13]. Within ST131, clade C is most strongly associated with multidrug resistance, in particular subclone H30R, which has acquired several mutations conferring resistance to fluoroquinolones [14]. In addition, almost all H30R isolates have resistance to third-generation cephalosporins conferred by the *bla_CTX-M-15_* gene, typically encoded on a plasmid [15, 16]. It is important to recognize that the high prevalence of *bla_CTX-M-15_* among clinical ST131 isolates is not a result of widespread horizontal transfer but due to the expansion of this highly successful subclone, which is characterized by high virulence, broad-spectrum resistance and low costs of plasmid carriage [16, 17]. This unholy but highly successful combination of traits has made ST131 one of the commonest *E. coli* genotypes in the clinic [18] and contributes to the disproportionate association of this subclade with sepsis [13]. Overall then, ST131 makes up a large proportion of clinical *E. coli* isolates with extended-spectrum beta-lactamases (ESBLs) [19], which are in turn responsible for a high proportion of MDR infections and UTIs [20, 21].

The concept of selected decolonization of undesirable bacterial genotypes is not a novel one. Historically, decolonization has used nonabsorbable antibiotics such as colistin to decrease the risk of infection by Gram-negative pathogens, for example in intensive care patients [22–25]. However, if decolonization is to be used as a sustainable strategy for antimicrobial stewardship, its reliance on the use of antibiotics is undesirable. More recent strategies have exploited the narrow host range and self-propagating characteristics of bacteriophage to selectively remove MDR pathogens from the intestinal reservoir [26]. Phage, for example, has been used to remove persistent carbapenemase-producing *Klebsiella pneumoniae* [27]. Phage applications often cause rapid evolution of resistance [28] and it remains to be seen whether bacterial phage resistance will preclude the widespread use of phage in decolonization of highly prevalent MDR lineages such as *E. coli *. Nevertheless, because so many dangerous clinical infections in humans that are caused by *E. coli* originate from genetically similar ST131 isolates, this group of closely related bacteria present a highly promising target for selective removal by strain-specific phage [29].

Decolonization of MDR microbes has also been attempted using probiotic non-pathogenic micro-organisms [30–32], and this strategy has displayed great promise in some cases [33]. Here, competition for nutrients such as iron, or antagonism based on the production of antimicrobial peptides such as bacteriocins, is the basis for displacement; both are believed to contribute to the efficacy of the probiotic strain *E. coli* Nissle [34, 35]. While there have been promising results from the use of the probiotic *E. coli* 83 972 to prevent serious infections in the bladder [30, 36, 37], several studies have failed to demonstrate effective decolonization or reduction in carriage of drug-resistant bacteria using probiotics in the guts of pigs and humans [32, 38, 39]. These results fit with the broad conclusion that the clinical efficacy of probiotics is highly variable [40, 41].

The production of bacteriocins, specifically microcin M and H47, has been reported as being central to the success of probiotic strain *E. coli* Nissle (Nissle) in competition [35], while bacteriocin production is believed to be one of the main mechanisms of antagonistic suppression of pathogens by probiotics [42–44]. Competitive displacement using probiotics is therefore likely to be limited by ecological barriers in diverse communities such as the gut. For example, a normal, healthy gut is composed of bacterial niches that are already filled [41]. Even if probiotics can suppress competitors *in vitro* [45]*,* they are likely to be at a frequency-dependent disadvantage when ingested in the gut, especially if resident and probiotic produce different suites of bacteriocins. This is because the competitive advantage conferred by the production of bacteriocins is dependent on the number of producers, since the concentration of secreted bacteriocins increases with the prevalence of producers [46, 47]. From the perspective of decolonization, more abundant resident microbes should be better able to defend their niche against invaders [48]. We would therefore predict that the efficacy of probiotic-based competitive displacement is likely to be limited by positive frequency-dependent fitness.

The ability of bacteriocin producers to defend niches and suppress invasion means that a combination of phage and ingested probiotics could be particularly powerful [49]. When bacterial hosts are at a high initial density, more phage infections will take place and therefore more progeny phage will be produced, leading to more rapid transmission. If phage can rapidly decrease the population size of an MDR target this could create the conditions under which a probiotic strain can invade a vacated niche and subsequently suppress or displace the target microbe via frequency-dependent competition. Other synergies could arise if competitive interactions increase the fitness costs of phage resistance [50] or if indirect effects reduce the benefits to be gained from phage resistance [51]. Finally, phage/probiotic combinations that effectively suppress the population size of targets could reduce mutation supply and therefore slow the rate of evolution of MDR strains to subsequent challenges from hosts or clinical interventions [52].

Here, we aimed to explore the evolutionary ecology of these interactions *in vitro* using a strain of human clinical interest. We tested the efficacy of competitive displacement of a well-characterized H30R clone of ST131 (EC958) with a conspecific probiotic: *E. coli* Nissle, which is the active ingredient of a widely used probiotic treatment [53]. In particular, we investigated whether the addition of phage and probiotics can break the frequency-dependent dominance of a resident target. We also tested whether we could see effective phage/probiotic-based decolonization in a range of environmental conditions (anaerobic, aerobic, presence/absence of a diverse community). Finally, we explored whether phage–probiotic combinations lead to stable suppression or elimination of a target by measuring the effect of phage on the longer-term dynamics of within species competition. We also assessed the impact of the competitor on the evolution of phage resistance and its associated fitness costs.

## Methods

### Culture and enumeration of bacteria and phage

The *E. coli* ST131 strain EC958 was kindly provided by Professor Matthew Upton of the University of Plymouth and was originally sourced from a patient in the UK in 2005 presenting with a UTI [54]. The *E. coli* Nissle 1917 strain was sourced from Mutaflor capsules (supplied by Linda Versand Apotheke, Germany). A diverse collection of wild-type *E. coli* and *Escherichia marmotae* with a range of serotypes and phylogenetic characteristics were also used in host range testing [55, 56] (Table S1). All liquid cultures were performed in Lysogeny Broth (LB) incubated with shaking at 180 r.p.m. at 37 °C, unless otherwise stated. Culture-based methods were used to assay the relative frequencies of Nissle and ST131 in competition or in transfer experiments by using serial dilution and plating out on LB agar (without antibiotic) and on LB agar containing 5 µg ml^−1^ of cefotaxime or 30 µg ml^−1^ of nalidixic acid in experiments with complex communities. Cefotaxime or nalidixic acid were used to distinguish between the ST131 and Nissle colonies as ST131 is known to be resistant to both antibiotics. During serial transfer experiments, the frequencies of ST131 and Nissle were also estimated by plating on *E. coli* coliform ChromoSelect agar (Merck, Darmstadt) containing novobiocin at 5 µg ml^−1^ (hereafter ChromoSelect agar). On this medium Nissle colonies are large and dark blue, while ST131 colonies are small and purple (Fig. S1, available in the online version of this article). Colony morphology was used in preference to selective plating using antibiotic resistance when proportions of ST131 were greater than 0.1 as this minimizes measurement errors.

Phage concentrations [in plaque-forming units (p.f.u.) ml^−1^) were measured by titration on a lawn of sensitive bacteria. Briefly, samples were chloroform-extracted (chloroform : sample 1 : 10 v/v), followed by centrifugation for 25 min at 3500 r.p.m. at 4 °C. The supernatant was then resuspended in M9 buffer (22 mM Na_2_HPO_4_; 22 mM KH_2_PO_4_; 8.6 mM NaCl; 20 mM NH_4_Cl), and 10-fold serial dilutions were prepared in M9 buffer and then spotted (5 µl) on soft-agar (0.5 %) overlay plates containing ST131. Control treatments (without phage) were checked for absence of phage prior to starting experiments and p.f.u. ml^−1^ were recorded at each transfer in all phage treatments.

### Isolation of narrow host range phage

Phages infectious for *E. coli* ST131 were isolated from wastewater (Falmouth sewage treatment works, UK) and pig faeces (Healey’s Cyder Farm, Truro, UK), using an enrichment method. For each sample, 1 ml of the sample and 6 ml of LB were mixed and inoculated with 60 µl of an overnight culture of *E. coli* ST131 and incubated overnight in a shaking incubator; cultures were serially cultured daily by diluting 1 ml into 6 ml of fresh LB and adding 60 µl of a fresh overnight culture of ST131. After 24 h, 2 ml samples were removed for phage isolation, mixed with 400 µl of chloroform before undergoing centrifugation and filtration via 0.22 µm filters.

These samples were used in spot tests on soft-layer LB agar (0.5 %) of *E. coli* ST131 to isolate potential phages. The presence of clear plaques after incubation of the plate at 37 °C indicated that phages were present in the sample. To obtain single-phage genotypes, one or more rounds of plaque purification were performed by picking a single plaque from plaque assays and infecting a liquid culture of early-logarithmic growing *E. coli* ST131 (OD_600nm_ 0.2–0.3) in LB. To generate high-titre phage stocks, phage amplification was performed at a multiplicity of infection (m.o.i.) of 0.01 to *E. coli* ST131 with an OD_600nm_ 0.2–0.3 in LB, and phage was isolated after overnight incubation shaking at 180 r.p.m. and 37 °C, followed by chloroform extraction of phage as described above. Phage stocks were stored at 4 °C.

The infectivity and specificity of each amplified phage sample was assessed by performing spot tests on our panel of 16 *Escherichia* spp. isolates, which included ST131 and Nissle (Table S1). First, 5 µl of 10-fold serial dilutions of each amplified phage was spotted onto each LB soft-agar lawn containing a single bacterial strain and incubated overnight at 37 °C. The presence of plaques indicated that the phage did indeed infect the bacterial strain used. This led to the identification of two phages, D (vB_EcoP-CC1) and H (vB_EcoP-CC2), which were isolated from wastewater and pig faeces samples, respectively, that infected *E. coli* ST131 but not Nissle or other *E. coli* in our panel, and these phages were used in the subsequent competition experiments.

### Phage genome sequencing

#### Phage amplification and DNA isolation

Phage stocks were amplified to generate enough genetic material for Illumina sequencing. Two rounds of phage amplification were performed, followed by chloroform extraction as described above. To concentrate the resulting phage stock, polyethylene glycol precipitation was performed using previously published protocols [57] followed by concentration with 15 ml 100kDa-Amicon filters (Merck, Darmstadt) [58]. The Norgen Biotek Phage DNA Isolation kit was used to extract phage genetic material as per the manufacturer’s instructions. Samples were quantified and analysed for purity using Qubit and Nanodrop and by running on a 0.8 % agarose gel before being stored at −80 °C.

#### Phage DNA library preparation and sequencing

Library preparation and Illumina sequencing of purified phage DNA were carried out at the Centre for Genomic Research at the University of Liverpool. A total of 250 ng of DNA per sample was fragmented using mechanical shearing on a Bioruptor Pico sonication device, targeting fragments of ~350 bp (nine cycles of 15 s ON/90 s OFF – additional cycles were performed whenever needed). Fragmented DNA was then used as input material for the NEBNext Ultra II library prep kit for Illumina. We used fivefold diluted adaptor for the adaptor ligation step. Size selection using beads was adapted for 300–400 bp insert size. Following four cycles of amplification, the libraries were purified using AMPure XP beads and the concentrations measured by Qubit. Size distribution was assessed using the Bioanalyzer and equimolar pooling of the final libraries was performed. The final pool was cleaned up to remove primer dimers and adaptor leftovers. The quantity and quality of the pool were assessed by the Bioanalyzer and by qPCR using the Illumina Library Quantification kit from Kapa on a Roche Light Cycler LC480II according to the manufacturer’s instructions. Briefly, a 10 µl PCR reaction (performed in triplicate for each pooled library) was prepared on ice with 8 µl SYBR Green I Master Mix and 2 µl diluted pooled DNA (1 : 1000 to 1 : 100 000 depending on the initial concentration determined by the Qubit dsDNA HS Assay kit). PCR thermal cycling conditions consisted of initial denaturation at 95 °C for 5 min, 35 cycles of 95 °C for 30 s (denaturation) and 60 °C for 45 s (annealing and extension), melt curve analysis to 95 °C (continuous) and cooling at 37 °C (LightCycler LC48011, Roche Diagnostics Ltd, Burgess Hill, UK).

Following calculation of the molarity using qPCR data, template DNA was diluted to 12pM and denatured for 5 min at room temperature using freshly diluted 0.2 N sodium hydroxide (NaOH) and the reaction was subsequently terminated by the addition of HT1 buffer. To improve sequencing quality control 1 % PhiX was spiked in. The libraries were sequenced on the Illumina MiSeq platform (Illumina, San Diego, CA, USA), generating 2×250 bp paired-end reads.

#### Phage genome assembly and annotation

Raw fastq files were trimmed for Illumina adapter sequences using Cutadapt version 1.2.1 [59]. The option -O 3 was used, so the 3′ end of any reads that matched the adapter sequence for 3 bp or more were trimmed. The reads were further trimmed using Sickle version 1.200 (https://github.com/najoshi/sickle) with a minimum window quality score of 20. Reads shorter than 15 bp after trimming were removed. Prior to assembly the samples were subsampled to 50 000 paired reads with the command ‘seqtk sample -s100’ to prevent misassembly due to excessively high coverage. Both samples were assembled using the metaviralSPAdes mode of SPAdes with default parameters [60]. CheckV was run on the assemblies to assess genome completeness [61]. Genome annotation was performed using Prokka, with the options ‘-kingdom Viruses’ and ‘-addgenes’ [62].

Taxonomic information was derived by running the genomes through the vContact2 pipeline [63] to cluster the phages among close relatives, using the Prokaryotic Viral RefSeq 97 database as a reference. Family-level taxonomic assignment was based on the taxonomy of the other phages that clustered together (*n*=25), in all cases being either *Podoviridae* or *Autographiviridae* (a family of podoviruses). Both phage genomes (D and H) were of similar sizes (44531 and 44532 bp) and nucleotide identity was measured using the FastANI tool [64]. Trimmed reads were mapped reciprocally to each genome to identify genetic polymorphisms using the breseq pipeline (version 0.38.1) [65]. Phage genomes were screened for antimicrobial resistance genes and virulence factors through the ResFinder and VirFinder webserver [66].

### Competition experiments in aerobic conditions

To assess the effect of phage predation and within-species competition on the fitness of the MDR *E. coli* strain ST131, we set up a factorial experiment with phage and competition treatments. Competition treatments used four frequencies of the probiotic Nissle and ST131: Nissle-only control; 50 % ST131; 90 % ST131; and an ST131-only control. Phage treatments comprised a no-phage control, phage D only, phage H only and a combined D and H treatment. To initiate competitions, overnight cultures were diluted 100-fold and combined at appropriate ratios; and phage was added at an m.o.i. of 0.1. All treatments used 6 ml of LB in 30 ml glass universals and were incubated for 24 h in a 180 r.p.m. shaking incubator at 37 °C.

### Competition in anaerobic chambers

Here we aimed to test the hypothesis that phage and competitor-mediated suppression of ST131 would also be effective under anaerobic conditions. Competition experiments and precultures of individual strains were performed using 25 ml serum flasks with butyl rubber stoppers containing 5 ml LB, as described previously [67]. In brief, these flasks were set up as anaerobic chambers by using 50 µg ml^−1^ cystine (a deoxygenating agent) and resazurin at 1 µl ml^−1^ as a redox indicator to confirm anaerobic conditions. In addition to competition from Nissle, these experiments also explored the impact of competition from a diverse microbial community isolated from pig faeces. Isolation and storage of this community was as described previously [67]. The competition experiment used a full factorial design with two community treatments (community/no-community) crossed with bacterial (ST131 only, Nissle and ST131 and Nissle only) and phage treatments (no-phage control and phage H at two concentrations). The experiment was replicated four times for most treatments, with four additional replicates for the Nissle and ST131 treatment. The phage concentrations were 2×10^5^ and 2×10^6^ p.f.u. ml^−1^, equating to m.o.i.s of 1 and 10, respectively. Flasks were inoculated with initial bacterial concentrations of 2×10^5^ colony-forming units (c.f.u.) ml^−1^ of ST131 and identical concentrations of Nissle and/or pig community c.f.u. as dictated by experimental treatment and cultured for 24 h. Experiments were run over two transfers, diluting 100 µl into the new chamber at the second transfer. To analyse samples from these experiments, nalidixic acid and ChromoSelect agar were used for selective culture of ST131, as plating of anaerobic cultures of the pig community produced no colonies under these conditions (*n*=4).

### Serial transfer experiments in aerobic conditions

To assess how both phage predation and ecological competition interacted to affect long-term ST131 persistence and phage resistance, we carried out a 14 day serial transfer experiment. We used a full-factorial design (phage/no phage, Nissle/no Nissle) to explore the fitness and dynamics of ST131. Competition treatments used a 50 : 50 mix of Nissle and ST131, while phage was added at an m.o.i. of 0.1. Each treatment was replicated six times and ran initially for 7 days. Bacteria and phage were propagated aerobically in 6 ml LB in 30 ml universals, as described above. At each 24 h period, 60 µl from each replicate was transferred into 6 ml of fresh LB broth to refresh depleted nutrients. Cultures were sampled daily and stored at −80 °C in 30 % glycerol for subsequent analysis. To investigate the longer-term trajectory of bacterial and phage dynamics and to mimic clinical interventions using multiple phage doses, the experiment was restarted to run for a further 7 days using glycerol stocks taken from the final timepoint (T7) of the previous experiment. We divided the cultures that had previous been exposed to one round of phage: one replicate set was cultured without phage, while the second half received an additional phage dose (at m.o.i. 0.1) at day 8 (T8). Phage and bacterial densities were measured at T8, T11 and T14.

### Assay of phage resistance

Phage resistance assays were conducted at T4 and T7 of the selection experiment, as detailed above, to determine any change in the sensitivity of the evolving ST131 clones to phage H. For each replicate of all treatments, 48 individual colonies were picked from LB agar plates containing 5 µg ml^−1^ cefotaxime to ensure that the colonies tested were ST131 only. Each colony was cultured overnight in 200 µl LB in 96-well plates; cultures were then diluted 10-fold in M9 buffer. The following day 25 µl of a phage H culture (2–4 x 10^5^ p.f.u. ml^−1^) was used to prepare phage lines on square LB agar plates. Once dry, 12 colonies were streaked through each phage line and plates were incubated overnight at 37 °C. Ancestral stocks of ST131 and Nissle were also streaked as controls. Resistance and susceptibility were scored as 1 and 0, respectively, whereby resistance was indicated by continued bacterial growth where it crosses the phage line, whereas susceptibility was indicated by a reduction in bacterial growth where the phage line was crossed.

### Fitness of phage-resistant mutants

We used competition experiments to see how the 14 day transfer experiment may have affected the relative fitness of evolved *E. coli* ST131 and to quantify any fitness costs that may be pleiotropically associated with phage resistance. We isolated a rifampicin-resistant mutant from our ancestor *E. coli* ST131 by spreading dense overnight cultures onto LB agar containing 50 µg ml^−1^ rifampicin and streaking out strongly growing colonies. Rifampicin-resistant mutants are commonly less fit than their wild-type counterparts so we measured competitive fitness in an invasion design. We isolated a single clone from each independent lineage and initiated a competition experiment with a 9 : 1 ratio of the rifampicin-resistant ancestor and an evolved clone, based on optical density (OD_600nm_) of overnight cultures. We measured competitive fitness over two transfers, carried out as per the serial transfer design above. The fitness experiment used four replicate cultures of each evolved lineage and eight replicates of the marked ancestor versus its wild-type counterpart. Dilution plating of the second transfer on LB without antibiotic and with 50 µg ml^−1^ rifampicin allowed us to quantify the final proportion of evolved clones. Relative fitness was calculated based on the changing proportion of evolved clones in competition (see below).

### Statistics

Initial and final proportions of ST131 (or other mutants of interest) were used to calculate the relative fitness in competition using the following expression: fitness = [*x*1 × (1 – *x*0)] / [*x*0 × (1 – *x*1)], where *x*1 is fraction of ST131 (or the clone of interest) at T1 and *x*0 is fraction of ST131 at time zero [68]. Dynamics during serial transfers used mixed model using the lmer function in the lme4 package, with replicate included as a random effect to account for the repeated measurements made on independent lineages [69].

## Results

### Isolation and characterization of phage

Host range testing identified two phages (D and H) that could infect *E. coli* ST131 isolate EC958, but not Nissle or any of the other *Escherichia* sp. isolates in our panel. lllumina sequencing identified both phages D and H as members of the family *Autographiviridae*, with a closest blast hit to *E. coli* virus LL11 [70]. These are double-stranded DNA phage, described as lytic in earlier work [71], which is consistent with the clear plaques and effects on bacterial density seen here. Both phage genomes (D and H) were nearly identical in size (44 531 bp and 44 532 bp, respectively) and sequence (nucleotide identity >99.99 %), with 52 estimated ORFs. Reads from each phage were cross-mapped to the other phage genome assembly to identify polymorphisms. Two single-nucleotide polymorphisms (SNPs) were identified between the phages and a blast search of the relevant phage D genes identified the genes as a tail fibre protein (locus *FCJLANJD_00018*) and a hypothetical protein (locus *FCJLANJD_00044*). While either or both may contribute to the observed differences in phenotype, tail fibres are frequently linked to host recognition and adsorption. No predicted antimicrobial resistance or virulence genes were identified following screening with the ResFinder and VirulenceFinder webservers. Both phage genomes have been submitted to ENA (study accession number PRJEB59323).

### Frequency-dependent competition in aerobic culture

**Fig. 1.**
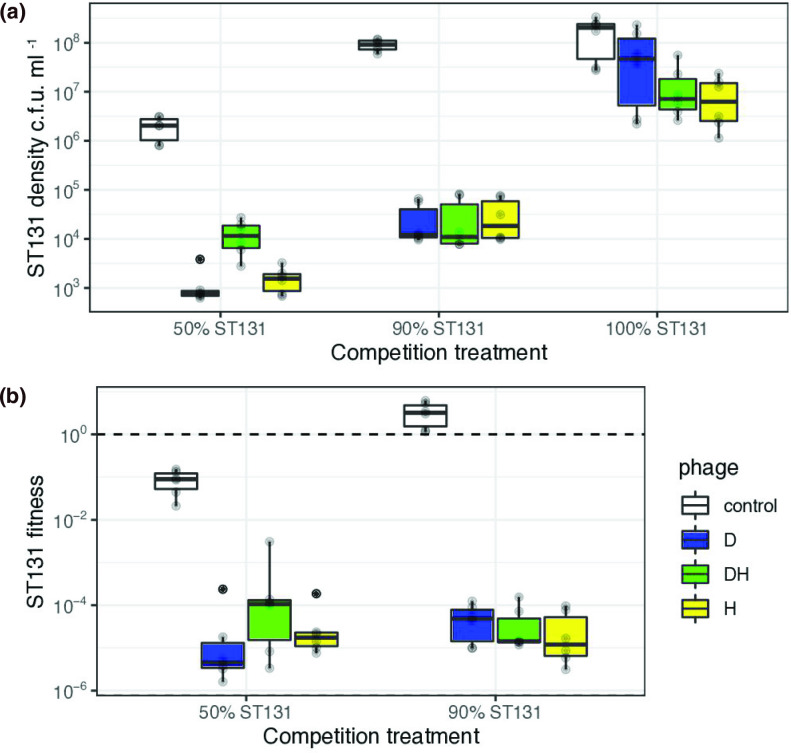
Effects of within-species competition and selective phage predation on suppression of multidrug-resistant *E. coli* ST131 during 24 h of aerobic culture. Experiments used two strain-specific phages in single strain cultures (100 % ST131) and in competition with the probiotic *E. coli* Nissle strain at two frequencies of ST131 (50 and 90 %). Data are (**a) **boxplots of six replicates of culturable counts of ST131 and (**b)** relative fitness in competition with Nissle. Relative fitness was based on the change in proportion of ST131 over the course of the experiment (see Methods) - the dashed horizontal reference line indicates equal fitness of competing bacteria. Boxplots show the median and interquartile range; solid black circles are outliers and semi-transparent circles are the remaining raw data.

Having identified ST131-specific phages, we wanted to test the ability of these phages D and H to suppress ST131 density, and whether this suppression ability was dependent on the presence of a competitor (Nissle), which is a probiotic strain that was not susceptible to the phages used in this experiment (Fig. 1a). This shows that each of the single phages could significantly repress ST131 abundance, but only by approximately one order of magnitude. In contrast, phage in the presence of the Nissle competitor reduced ST131 abundance by three–four orders of magnitude (phage and competition treatments interaction *F*_4,45_=21.2, *P*<0.0001, Fig. 1a). The different phages (D, H) did not have different effects on density over a single transfer, as pooling of these treatments resulted in no significant loss of explanatory power in our statistical model (*F_3,48_* =2.44, *P*=0.08).

Predation by ST131-specific phages decreased the fitness of ST131 when in competition with Nissle (Fig. 1b, *F*_1,33_=282, *P*<0.0001). Based on the theory of bacteriocin-mediated competition, we also hypothesized that we would see frequency-dependent fitness of ST131 in competition with Nissle and this was confirmed with our first set of competition experiments: ST131 fitness increased with its frequency in mixed culture (Fig. 1b, *F*_1,32_=16.0, *P*=0.0004). Critically, this meant that Nissle could not outcompete ST131 in the absence of phage if ST131 was at high frequencies at the start of experiments (Fig. 1b). We also observed a strong interaction between phage and competition treatments: phage reduced the fitness of ST131 much more substantially when ST131 was at high initial frequency (*F_2,30_* =8.21, *P*=0.0017), confirming that the combination of phage and within-species competition has the potential to suppress an abundant and prevalent target genotype.

A short serial transfer experiment over 4 days tested whether the phage combination of D and H could suppress the evolution of phage resistance, and whether phage alone could suppress ST131 density after more than one transfer. Phage H gave improved suppression of ST131 in monoculture when compared to phage D over this longer time period (phage treatment and time^2^ interaction *F_3,108_* =3.28, *P*=0.024; phage treatment and time interaction *F_3,111_* =5.16, *P*=0.0023, Fig. S2). Use of phage D resulted in more rapid evolution of resistance when compared to phage H or the DH cocktail (glm with quasibinomial error *F*_2,33_=6.03*, P*=0.006*, n*=140 streaks, Fig. S3). This experiment also revealed strong cross-resistance between phages D and H and no benefit from use of a cocktail (Figs S2 and S3). For these reasons, only phage H was used in subsequent experiments.

### Competition in anaerobic chambers

**Fig. 2.**
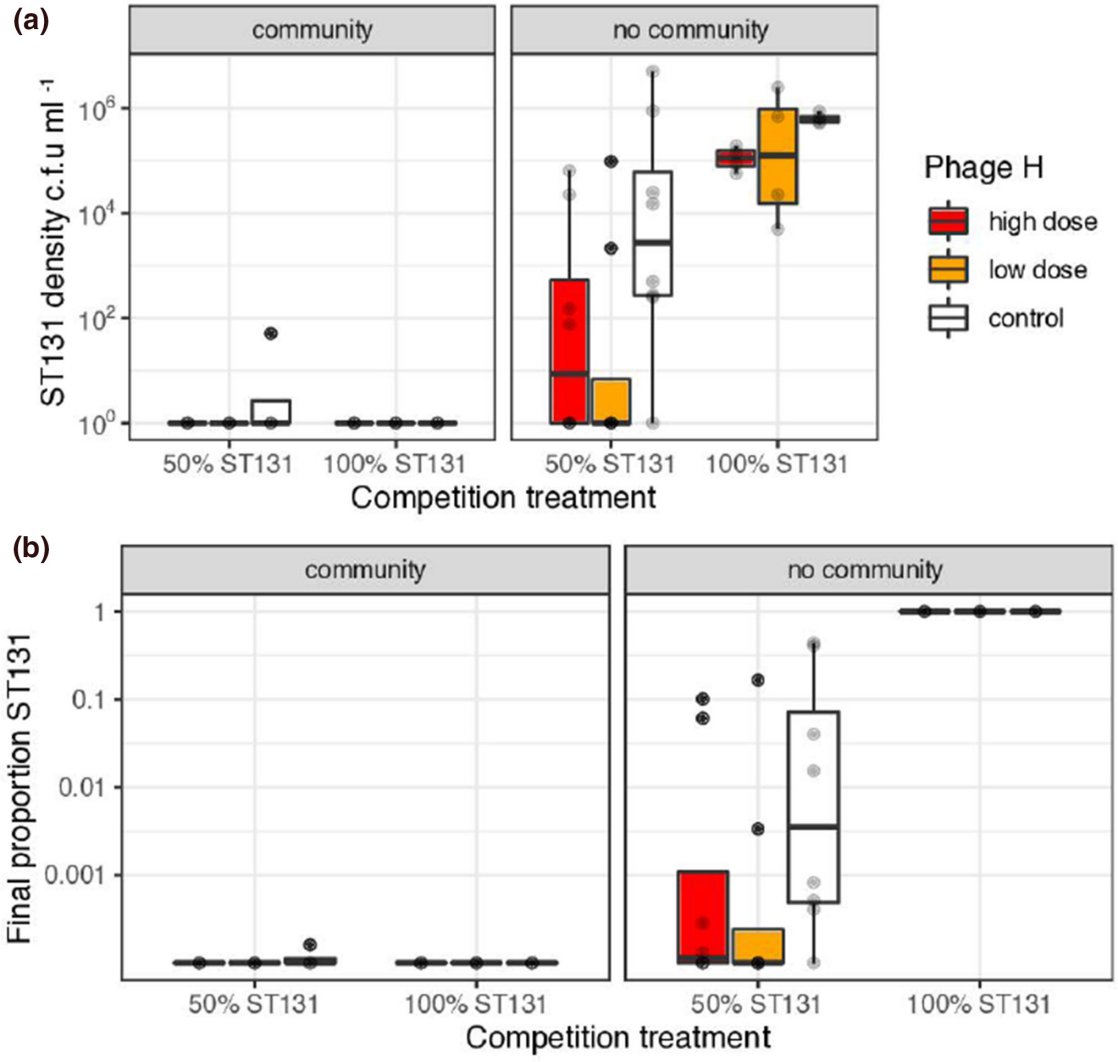
Effects of within-species competition and selective phage predation on suppression of multidrug-resistant *E. coli* ST131 during two passages in anaerobic culture. Experiments used strain-specific phage H at two doses (m.o.i.s of 1 and 10 in low and high doses, respectively) plus a phage-free control. Competition was manipulated with the presence/absence of the probiotic Nissle (50 % ST131/100 % ST131) and by the presence/absence of a pig faecal community (community/no-community panels). (**a)** Boxplots of eight replicates showing final density. (**b)** Final proportion of ST131 transformed by adding +0.0001 to allow display of zeroes on a log scale.

Here we aimed to test whether phage- and competitor-mediated suppression of ST131 would also be effective under anaerobic conditions, which are more reminiscent of gut conditions where *E. coli* commonly resides [72]. In anaerobic conditions ST131 was very sensitive to competition from a mixed bacterial community, as almost no resistant colonies were recovered from experimental competitions involving a pig faecal community (Fig. 2). All formal statistical analyses examined experiments using phage applied to cultures without communities. As above, within-species competition and phage acted together to reduce densities of ST131 (Nissle effect *F_1,34_* =26.8, *P*<0.0001, phage effect *F_2,432_* =4.15, *P*=0.025, Fig. 2a). We did not see a significant interaction between phage and Nissle competition, suggesting that phage and competition acted independently and additively (*F_2,30_* =0.73, *P*=0.49), although 6/8 replicates from the treatment with low-m.o.i. phage and Nissle competition excluded ST131 to densities below our detection limits (25 c.f.u. ml^−1^; Fig. 2a). Overall, within- and between-species competition drove ST131 down to undetectable levels in so many replicates that analyses of relative fitness would have been unhelpful (Fig. 2b).

### Serial transfer competition experiments

**Fig. 3.**
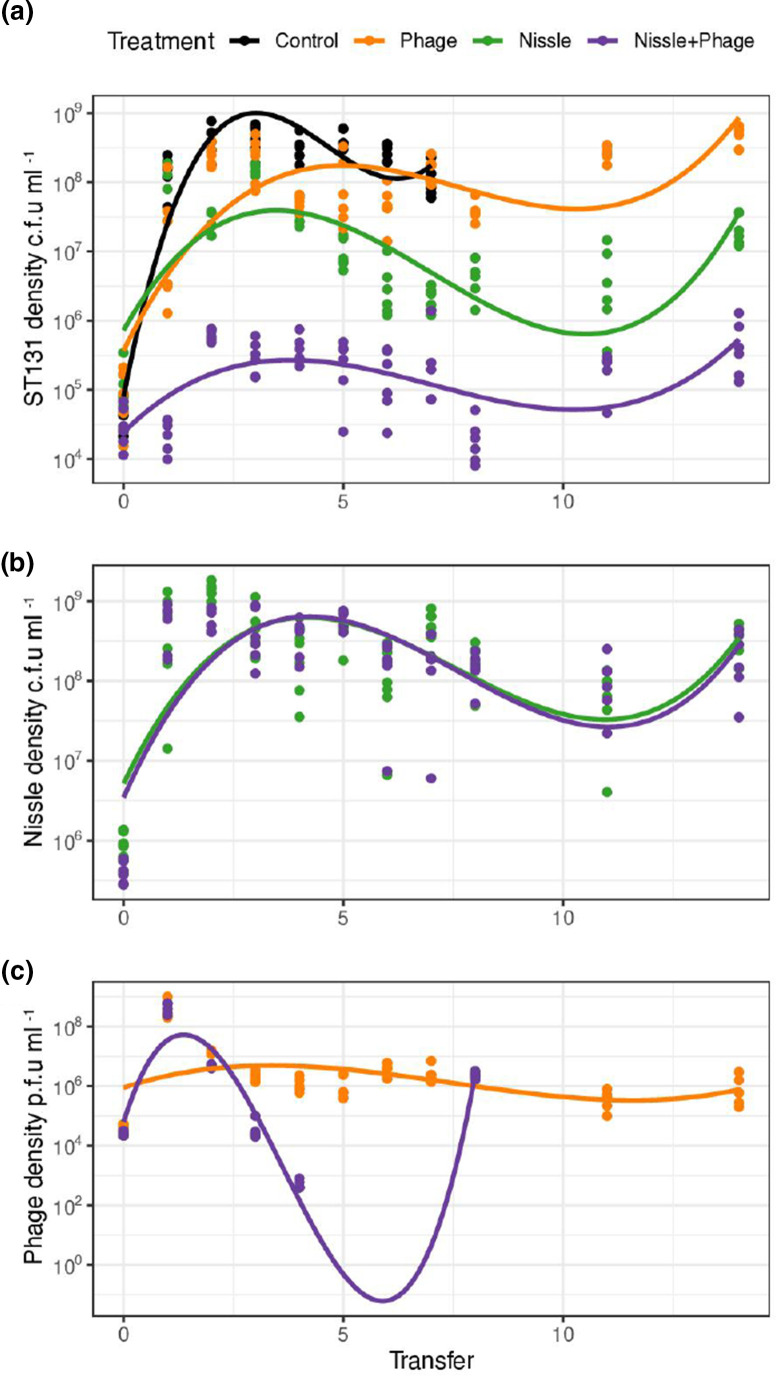
Population dynamics of ST131, Nissle and phage H over the course of a 14 day transfer experiment. (**a)** The log_10_ density of ST131 changing when in competition with Nissle, when exposed to phage H and when exposed to both Nissle and phage H simultaneously. For phage-containing treatments, additional phage was added at T8. The ST131-only control was only run for the first 7 days of the experiment. Fitted lines show polynomial regressions. (**b)** The log_10_ density of Nissle changing when in competition with ST131 only and when in competition with ST131 in the presence of phage H. (**c)** The log_10_ density of phage H changing when applied to pure ST131 cultures (phage treatment) and when also in the presence of Nissle (Nissle+phage). Data points are raw values for six replicates and fitted polynomial regressions for each treatment. Note that phage were at undetectable densities in the Nissle+phage treatment at transfers 5–7 and on transfers 11 and 14.

The experiments above only considered the dynamic of phage and bacteria over one or two transfers. In this 14 day transfer experiment, we sought to understand competitive dynamics over a longer time period. We analysed the effect of the presence of Nissle and phage H on the dynamics of ST131 using linear models. The overall variation between transfers could be captured with simple quadratic and cubic terms. We then used model simplification to explore the impact of our key experimental treatments (addition of phage and the competitor Nissle). Nissle had a significant effect on the density of ST131 (Fig. 3a, likelihood ratio test, *df*=1, $\chi^{2}$ = 44.3, *P*<0.0001), as did phage H (Fig. 3a, likelihood ratio test, *df*=1, $\chi^{2}$ = 24, *P*<0.0001). Importantly, the addition of phage H alone had a relative weak effect on reducing the density of ST131 (reducing log_10_ density by 0.25), and this was largely eroded by transfer 7. Phage had a much stronger impact on reducing ST131 density when Nissle was present (Fig. 3a, likelihood ratio test, phage X competition, *df*=1, $\chi^{2}$ = 48.0, *P*<0.0001).

At the first time point (T11) that phage densities were measured following the restart of the experiment, phage had dropped below detectable limits, a pattern consistent with extinction in the presence of resistant hosts (Fig. 3c). Despite this, the density of ST131 did not recover to the levels seen in the Nissle-only treatment, even though phage predation was undetectable at the end of this experiment. As might be predicted from host range data and earlier experiments, the addition of selective phage H had no impact on the density of Nissle (Fig. 3b, likelihood ratio, *df*=1, $\chi^{2}$ = 0.2, *P*<0.05). In contrast, Nissle did have a significant effect on the log_10_-transformed density of phage (Fig. 3c, Likelihood ratio test, *df*=1, $\chi^{2}$ = 8.1, *P*<0.01).

We added a second dose of phage to one set of replicates on restarting the experiment over days 8–14; we hypothesized that this might achieve some additional reduction in density if bacteria were partially resistant. The second addition of phage had no impact on bacterial densities over the second half of the serial transfer experiment for ST131 (phage-only treatments, *t*=−0.018, *df*=34, *P*>0.05; phage+Nissle treatments, *t*=−1.15, *df*=34, *P*>0.05), indicating that bacterial resistance to phage H was very effective. The second addition of phage did have a significant effect on the p.f.u. ml^−1^ of phage at T8 but was undetectable thereafter (two-sample *t*-test, phage-only treatment, *t* =-3, *df*=34, *P*<0.05; phage+Nissle treatments, *t*=−3.83, *df*=34, *P*<0.05). All phage treatments are therefore represented in all analyses and figures from T7 onward by the replicates that are exposed to additional phage.

### Relative fitness of ST131

**Fig. 4.**
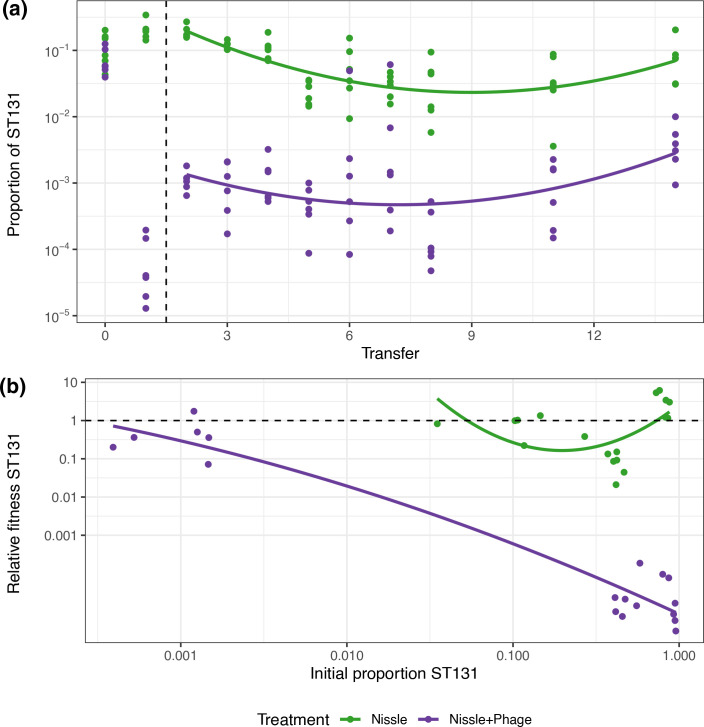
Fitness of ST131 over a 14-transfer experiment when in competition with the probiotic Nissle. ST131 was passaged in the presence and absence of phage H. (**a)** The proportion of ST131 (raw data) at each transfer and in the initial mixtures at T0. Lines show quadratic models fitted to all data from transfer 2 onward (i.e. after the vertical dashed line). (**b)** The relative fitness of ST131 based on the change in proportion of this genotype with respect to the set-up at T0, again with fitted quadratic model. The dashed horizontal reference line indicate equal fitness of competing bacteria. s equal fitness of competing bacteria.

The densities of ST131 had dramatic changes after the first transfer, especially in the phage addition, but thereafter settled into more regular dynamics. We analysed the dynamics from transfer 2 to transfer 14 of single phage addition and phage-free control treatments with linear mixed effect models. We detected slightly different trajectories in the proportion of ST131 across these treatments. In the Nissle-only treatment ST131 frequencies tended to decline from T2 onward, while after phage treatment frequencies increased gradually from T2–T14 (phage treatment transfer interaction, χ^2^=6.34, *df*=1, *P*=0.012, Fig. 4a). Across both, however, there was an uptick in ST131 frequency at the end of this experiment, especially in the final transfer (quadratic term χ^2^=20.6, *df*=1, *P*<0.0001, Fig. 4a). We saw qualitatively similar results using generalized linear models that pooled the single phage application and the treatment with two applications (phage X transfer interaction, *F_1,121_* =7.13, *P*=0.009; quadratic term, *F_1,122_* =32.3, *P*<0.0001).

A key prediction of this study was that the presence of phage would alter the frequency-dependent competitive interactions between Nissle and ST131. The competitive dynamics of ST131 and Nissle were clearly very different in the presence and absence of phage. Serial transfers showed that phage led to the persistence of ST131 at much lower frequencies, suggesting that phage altered the fitness equilibrium of ST131 when competing with Nissle. To explore this formally we used data from the 24 h transfer experiments (described in Fig. 1) and the serial transfer experiment to investigate fitness over a greater range of frequencies. To avoid pseudo-replication we used a randomly selected 24 h transfer (from days 1–7) for each independent replicate to test for different patterns of frequency-dependent fitness in the presence and absence of phage. In the absence of phage, the fitness of ST131 was lowest at intermediate frequencies (Fig. 4b). Frequency-dependent fitness was non-linear and was effectively described by a quadratic model using frequency^2^, a term which interacted with the presence of phage (phage treatment frequency^2^ interaction: *F_1,32_* =14.6, *P*=0.0006). In the absence of phage, ST131 could outcompete Nissle at high frequencies, had lower fitness than Nissle at intermediate frequencies but then reached equal fitness with Nissle when at just under 10 % frequency (Fig. 4b). In contrast, in the presence of phage, ST131 had robust negative frequency-dependent fitness, with lowest fitness at high frequencies. With phage present, ST131 reached equal fitness with Nissle when making up approximately 0.1 % of the population (Fig. 4b).

### Evolution of phage resistance

**Fig. 5.**
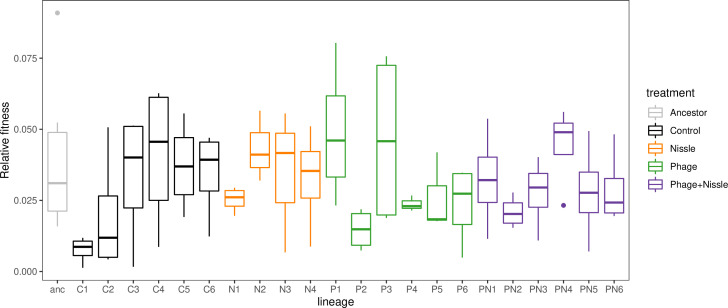
Relative fitness of end point lineages after exposure to phage and/or within-species competition from *E. coli* Nissle in a 14-transfer experiment. Note that resistance to phage is near fixation in phage-exposed lineages. All relative fitness is calculated from invasion experiments using an isogenic rifampicin resistant mutant derived from the ST 131 ancestor.

To investigate whether resistance was less likely to evolve or would evolve at a slower rate when exposed to phage and Nissle combined, resistance assays were conducted at T3 and T7 of the serial transfer experiment for the phage-only and the phage+competitor treatment, using 48 clones for each of the 6 replicates. At both time points, resistance was close to fixation in all replicates tested (98–100 %, data not shown) from both treatments and there was no evidence that within-species competition with Nissle affected phage resistance (two sample *t*-test, *t*=0.85, *df*=22, *P*>0.05). We also explored how serial transfer and near fixation of resistance would affect relative fitness of ST131 after exposure to phage and or Nissle. Inspection of fitness data shows that the fitness of clones from nearly all evolved lineages was very close to that of the ancestor (Fig. 5). Linear mixed effect models showed no effect of exposure to phage or Nissle on relative fitness (likelihood ratio tests χ^2^=0.001, *df*=1, *P*=0.97, χ^2^=0.14, *df*=1, *P*=0.71 respectively; Fig. 5), indicating that if there were any fitness costs associated with phage resistance then they were too small to detect with competition experiments.

## Discussion

As we predicted, *in vitro* suppression of a drug-resistant target by competition from a probiotic acting alone was only effective over a limited range of frequencies. The patterns of frequency-dependent suppression in this study, with the lowest fitness of ST131 at intermediate frequencies, are consistent with theoretical expectations of bacteriocin-mediated antagonism [73–75], and the known mechanism of microcin-based suppression of competitors in the Nissle strain [35]. This kind of dynamic could explain why the deployment of probiotics alone in decolonization is often ineffective [32, 38, 39, 49]: resident drug-resistant genotypes will be difficult to dislodge, as they are likely to have a numerical and physiological advantage over any ingested probiotic.

Importantly, phage alone could also not provide long-term suppression of ST131. In the absence of within-species competition the evolution of phage resistance meant that our MDR target could return to pre-phage densities within 20 generations (2 transfers). Phage resistance has also proved a barrier to the use of phage alone in other decolonization experiments *in vitro* [29]. As found in experiments with pig-associated *E. coli *, the combined action of phage and probiotic was essential for decolonization *in vitro* [49]. Despite evolution of phage resistance, we saw long-term suppression of *E. coli* ST131 with combined use of phage and probiotic, suggesting that experimental interventions had shifted mixtures of *E. coli* ST131 and Nissle to a new stable equilibrium. In contrast to previous studies, we did not see elimination of our MDR target to below detectable levels [49]. Analysis of frequency-dependent fitness and the stability of ST131 in the serial transfer experiment suggests that ST131 and Nissle co-exist in the presence of phage when ST131 is at a low but detectable frequency of ~0.1 %.

The presence of phage in the early stages of competition appears to exert a lasting effect on the fitness of the ST131 population. As the resistance phenotype is established throughout the ST131 population, phage H is rapidly driven to extinction. With phage predation undetectable, we would expect the frequency of ST131 to increase to the level seen in the Nissle-only treatment, if predator–prey interactions were the sole factor driving ST131 population size, but this is not what we observed. The evolution of resistance could provide a reasonable explanation for this, considering the widely documented fitness costs associated with resistance [76]. However, the persistence of phage-resistant ST131 at low frequencies, as well as the absence of a competitive cost of phage resistance in the absence of phage, suggests that this fitness cost is not straightforward. One possibility is that resistance to phage H increases the sensitivity of ST131 to the bacteriocin antimicrobials secreted by Nissle. Similar patterns have been observed for phage-resistant *Ralstonia solanacearum* and sensitivity to antimicrobials produced by *Bacillus amyloliquefaciens* [77]. Not only can antimicrobial efficacy be increased in the presence of phage [78, 79], but phage resistance is known to be able to increase the sensitivity of bacterial to diverse classes of antimicrobials [77, 80].

In the absence of phage, *E. coli* ST131 persisted at a higher frequency (approximately 1 % of the total population) during our 14-transfer experiment. The fitness of ST131 was lowest at intermediate frequencies and fitness started to increase at frequencies below 10 %. A stable ratio of 1 : 99 of ST131 : Nissle implies that fitness becomes approximately equal when the ST131 strain is at a frequency of approximately 1 %. This pattern mirrors previous work on bacteriocin production in *Pseudomonas aeruginosa,* in which serial transfers in broth culture led to a low stable frequency of bacteriocin non-producers [47]. In contrast, *E. coli* colicin producers in streptomycin-treated mice can drive sensitive non-producers to extinction [81]; similar dynamics are seen in competition in well-mixed broth [46, 48]. While coexistence of colicin producers and non-producers can occur in spatially complex environments [46, 48], this is unlikely to be the mechanism for co-existence of *E. coli* antagonists in broth culture. One explanation for stable co-existence of bacteriocin producers and non-producers is ‘competition-sensing’ [82]. The production and secretion of bacteriocins is metabolically expensive [83, 84]. Conditional expression of bacteriocins that is dependent on detection of competitors is the key idea behind competition sensing [82]. If competitors are rare then the cost of production may be greater than the benefits gained through very modest increases in access to resources. Inactivation of bacteriocin production when competitors are at low frequency could therefore allow the persistence of sensitive genotypes in mixed culture. Stress responses are known triggers for both microcin and colicin production [48, 85, 86], so frequency-dependent DNA damage resulting from microcins secreted by competitors is a plausible mechanism for the dynamics seen in this experiment.

In this study, low-cost resistance to phage emerged readily. In terms of improving the efficacy of phage-probiotic combinations, there are obvious gains to be made by minimizing the evolution of resistance to phage. There are a range of strategies for delaying the onset of phage resistance, and these can include simply screening phage *in vitro* and exploiting phage isolates that are less prone to resistance. The evolution of resistance can be delayed via the use of phage cocktails [87–89], in particular those that consist of phages that target different host receptors as resistance would require the accumulation of mutations in multiple target receptors. Broad-spectrum resistance is known to evolve in response to such phage cocktails, e.g. through mutating gene encoding global regulators, but such mutations are often associated with substantial fitness costs [90, 91]. Choice of microbial competitors is likely to be important for the efficacy of probiotic–phage combinations. Other work has sought to identify effective competitor bacteria based on the expression of microcins [49], but bacteriocin expression can correlate with virulence [92]. For human targets, a well-characterized probiotic such as Nissle is an excellent candidate, as its combines microcin expression with a low uptake of conjugative plasmids [93].

The selection of an effective microbial competitor for a MDR target must consider the ecological niche of the target and a realistic set of conditions for testing competitive ability. In this study, we found that phage and a probiotic competitor could displace ST131 in both aerobic and aerobic conditions, which is encouraging for the robustness of this methodology for a bacterium that is facultatively anaerobic. However, we also found that competition from a diverse community could readily displace *E. coli* ST131 in anaerobic chambers without the use of phage. This result could be interpreted as indicating that any increase in microbial diversity might help to suppress MDR bacteria. However, these results have to be considered in the light of the physiology of *E. coli *: although this species is a facultative anaerobe, it is a colonist of the gut epithelium, where oxygen diffusing from host tissues creates micro-aerobic conditions [94]. Respiration of oxygen has been shown to be vital for effective colonization of the gut in a mouse model by *E. coli* [72]. Facultative anaerobes such as *E. coli* are a minor part of the mammalian microbiome, comprising perhaps 1 in 1000 to 1 in 10 000 cells. Our results confirm that *E. coli* is a poor competitor with anaerobes in anaerobic conditions. A weaker competitive ability in the absence of oxygen is likely to restrict *E. coli* to this micro-aerobic niche [72], while *E. coli* is also dependent on anaerobic microbiota for providing simple sugars required for growth [72]. It follows that we should not look to anaerobes or nano-aerobic species (e.g. *Bacteriodetes fragilis*) as good candidates for the decolonization of MDR *E. coli *. These species will be present and are likely to already be limiting the colonization of some niches by *E. coli.* Good candidates for competitive decolonization should therefore either be conspecifics or facultative anaerobes, such as *Enterococcus faecalis *, that are able to occupy the same microaerobic epithelial niche.

The complementary use of probiotic bacteria to improve the therapeutic outcome of the use of phage is an example of adjunctive therapy [95]. Although there are many examples of the use of probiotics and phage as adjuncts to antibiotics, there is little evidence of their use to enhance the efficacy of one another [96, 97]. Research into the synergistic combination of antimicrobial agents is still largely dominated by examples of the use of alternative antimicrobials to improve the efficacy of antibiotics [98]. Turning our attention to the potential of other combinations of antimicrobials, such as probiotics and phage, will enable us to reduce our reliance on antibiotics and expand the diversity of our therapeutic arsenal against MDR pathogens [49, 99]. Since probiotics such as Nissle can use a range of mechanisms to benefit hosts or outcompete pathogens in more naturalistic growth conditions [45, 100], a clear next step is to explore these interactions in hosts.

## Supplementary Data

Supplementary material 1
