# Supplementary material for: Decolonizing drug-resistant E. coli with phage and probiotics: breaking the frequency-dependent dominance of residents
Source: Microbiology (Reading). 2023 Jul 7;169(7):001352. doi: 10.1099/mic.0.001352 (PMC10433417; doi:10.1099/mic.0.001352)

Supplementary Material for "Decolonizing drug-resistant *E. coli* with phage and probiotics: breaking the frequency-dependent dominance of residents."

Jessica Forsyth, Natalie L Barron, Lucy Scott, Bridget N J Watson, Matthew A W Chisnall, Sean Meaden, Stineke van Houte, Ben Raymond.

Table S1. List of bacterial strains used in this study

Name	species	serotype	source	description
EC958	*E. coli*		M. Upton, Plymouth	clinical ST131 isolate
Nissle 1917	*E. coli*		Mutaflor, Germany	widely used probiotic
K12	*E. coli*		ATCC	laboratory strain
REL606 (B)	*E. coli*		*E. coli* genetic stock centre	*E. coli* B – used in experimental evolution
tm8.6	*E. coli*	27	Medaney et al 2016	environmental isolate
tm4.1	*E. coli*	16	Medaney et al 2016	environmental isolate
A1.1	*E. coli*	8	Medaney et al 2016	environmental isolate
R3.4	*E. coli*	38	Medaney et al 2016	environmental isolate
R1.9	*E. coli*	5	Medaney et al 2016	environmental isolate
D2.2	*E. coli*	8		
A6.2	*E. coli*	5	Medaney et al 2016	environmental isolate
A6.5	*E. coli*	56	Medaney et al 2016	environmental isolate
A6.1	*E. coli*	38	Medaney et al 2016	environmental isolate
wc5.8	*E. coli*	12	Medaney et al 2016	environmental isolate
oc5.6	*E. coli*	12	Medaney et al 2016	environmental isolate
wc6.1	*E. coli*	27	Medaney et al 2016	environmental isolate
D7.8	*E. marmotae*	16	Medaney et al 2016	environmental isolate
oc5.1	*E. marmotae*	56	Medaney et al 2016	environmental isolate

Figure S1. Colony morphology of *E. coli* ST131 and Nissle strains on Ec Coliform Chromoselect Agar.

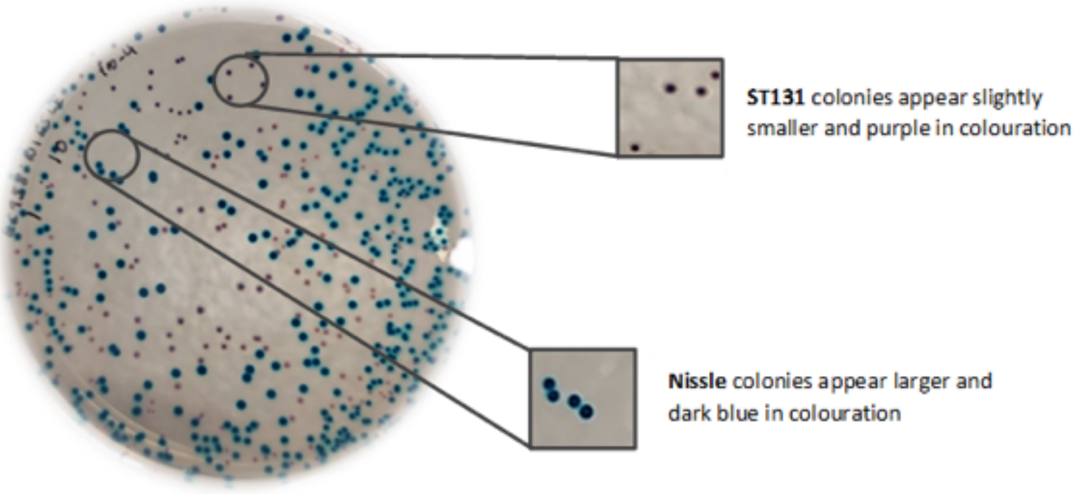

Figure S2. Dynamics of bacteria and phage in single strain infections over four transfers, here Nissle and ST131 densities are from separate replicates (*n* = 6). Treatments are bacteria only (control), in presence of phage D, phage H or in presence of combination of phage D and H. **A** shows bacterial densities with quadratic fitted models. In this experiment bacterial densities after treatment with phage D were not significant from controls. (Post hoc tests: main effect t – 0.7, P = 0.5 or in linear & quadratic interactions t = -0.3 P = 0.44 t = 0.06, P = 0.45). **B** shows phage densities in four transfers and demonstrates that none of the ST131 specific phages can persist in the presence of Nissle alone.

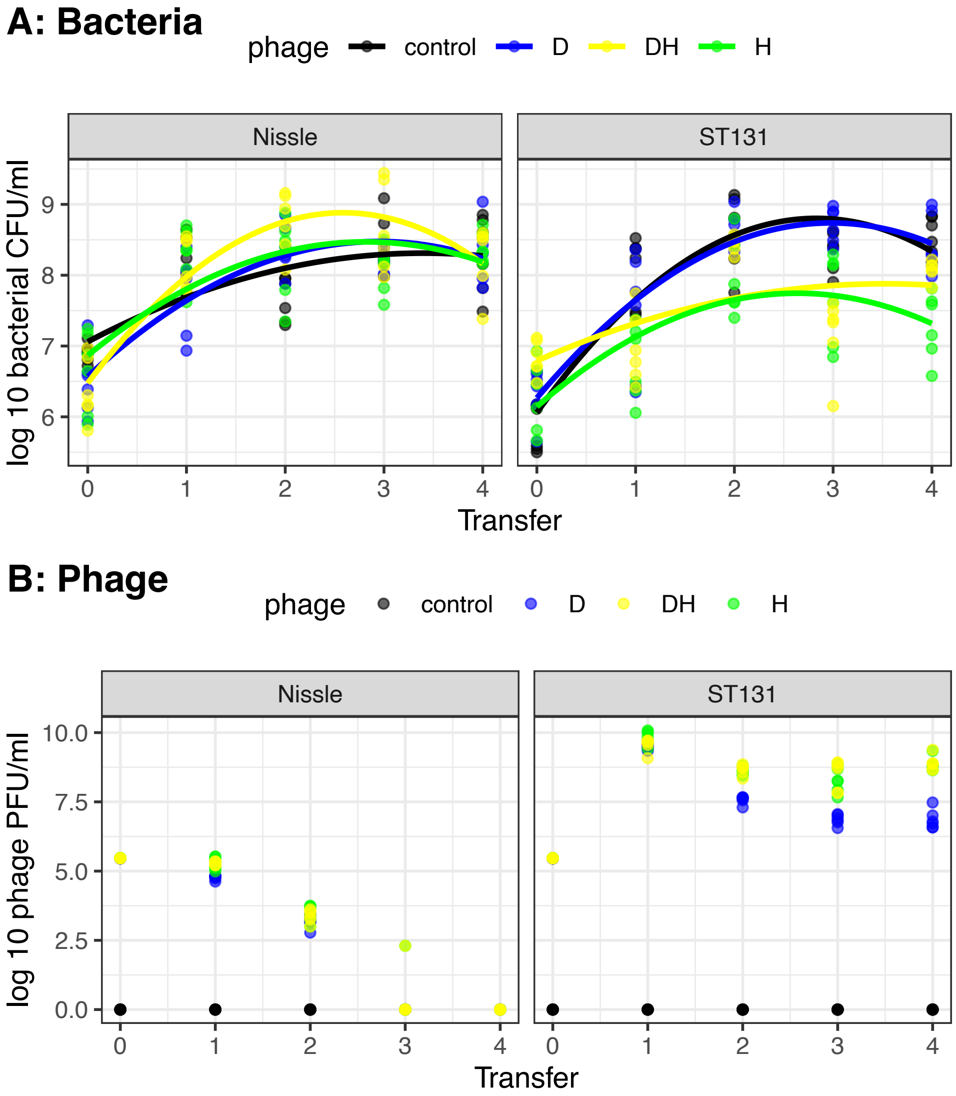

Figure S3. Evolution of resistance to phages D and H in a 4 day transfer experiment using monocultures of ST131. Data are boxplots of the proportion of resistant colonies from six replicates, with 24 colonies scored for each independent replicate. Data for resistance to phages D and H are presented in different panels.

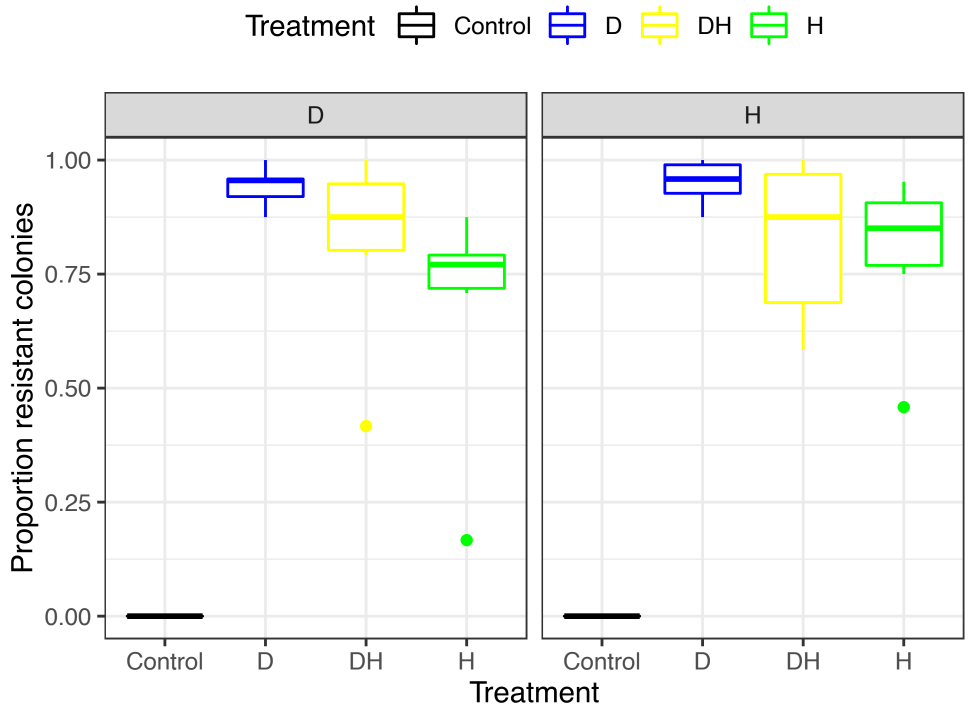

Supplement: Supplementary material 1 [file mic-169-1352-s001.pdf]
